# Bioengineered human skeletal muscle capable of functional regeneration

**DOI:** 10.1186/s12915-020-00884-3

**Published:** 2020-10-20

**Authors:** J. W. Fleming, A. J. Capel, R. P. Rimington, P. Wheeler, A. N. Leonard, N. C. Bishop, O. G. Davies, M. P. Lewis

**Affiliations:** School of Sports, Exercise and Health Sciences, Loughborough University, Loughborough, LE11 3TU UK

**Keywords:** Skeletal muscle, Regeneration, Tissue engineering, Satellite cell

## Abstract

**Background:**

Skeletal muscle (SkM) regenerates following injury, replacing damaged tissue with high fidelity. However, in serious injuries, non-regenerative defects leave patients with loss of function, increased re-injury risk and often chronic pain. Progress in treating these non-regenerative defects has been slow, with advances only occurring where a comprehensive understanding of regeneration has been gained. Tissue engineering has allowed the development of bioengineered models of SkM which regenerate following injury to support research in regenerative physiology. To date, however, no studies have utilised human myogenic precursor cells (hMPCs) to closely mimic functional human regenerative physiology.

**Results:**

Here we address some of the difficulties associated with cell number and hMPC mitogenicity using magnetic association cell sorting (MACS), for the marker CD56, and media supplementation with fibroblast growth factor 2 (FGF-2) and B-27 supplement. Cell sorting allowed extended expansion of myogenic cells and supplementation was shown to improve myogenesis within engineered tissues and force generation at maturity. In addition, these engineered human SkM regenerated following barium chloride (BaCl_2_) injury. Following injury, reductions in function (87.5%) and myotube number (33.3%) were observed, followed by a proliferative phase with increased MyoD+ cells and a subsequent recovery of function and myotube number. An expansion of the Pax7+ cell population was observed across recovery suggesting an ability to generate Pax7+ cells within the tissue, similar to the self-renewal of satellite cells seen in vivo*.*

**Conclusions:**

This work outlines an engineered human SkM capable of functional regeneration following injury, built upon an open source system adding to the pre-clinical testing toolbox to improve the understanding of basic regenerative physiology.

## Background

Skeletal muscle possesses an innate and robust capacity to regenerate following injury, with most injuries regenerating the tissue to a state indistinguishable from that prior to injury [1]. This regenerative capacity in vivo relies upon the presence of a resident stem cell population, satellite cells (SCs), which reside between the plasma membrane (sarcolemma) of muscle fibres and the encasing basement membrane [2–4]. SCs are characterised by the unique position they occupy within the tissue, but also the expression of the stem cell transcription factor Pax7 [5]. Following injury SCs are activated and proliferate readily [6], producing committed myogenic precursor cells (MPCs) marked by the presence of MyoD expression [7, 8]. MPCs then fuse together and with damaged muscle fibres, regenerating myofibres lost through injury [9–11]. In addition to a myogenic lineage, non-myogenic stem cells (fibro/adipogenic progenitors (FAPs)) and immune cells support regeneration by modifying the extracellular matrix and coordinating repair and regeneration [12–14]. The interactions between these additional cell types and MPCs have been shown to be vital in the regenerative process [15–17].

In severe muscle traumas such as volumetric muscle loss (VML), surgical trauma or partial muscles tears, the regenerative capacity of skeletal muscle can be overcome, leading to non-regenerative defects such as fibrosis [18–21], interstitial adipose accumulation [13, 22, 23] and heterotopic bone formation [24, 25]. In these circumstances, individuals are left with complications such as reduced function, increased chance of reinjury and debilitating pain [26–28]. In animal models of severe traumas, there has been limited success in manipulating the regenerative process to promote muscle regeneration and reduce non-regenerative defects. Although, where clear biochemical rationale exists, for example limiting the expansion of specific cell populations with small molecules, progress has been made [29–31]. This limited success may be attributed to the lack of easily manipulated, medium-throughput models of injury and regeneration, thus limiting understanding of the fundamental biology of muscle injury and regeneration/repair.

Due to the complex cell-cell interactions and three-dimensional (3D) environment necessary to accurately mimic skeletal muscle regeneration, models of muscle injury to date have been predominantly based around laboratory animals. However, animal models face limitations with low experimental throughput, complex genetic manipulations, complex pharmacological manipulation (compared to cell cultures) and ethical considerations, in addition to inherent biological variation from humans, and therefore there is a clear requirement to develop accurate and robust ex vivo models of pathophysiology [32, 33]. Advances in tissue engineering have made it possible to create engineered skeletal muscle to understand complex physiological phenomenon. Engineered skeletal muscles from cell lines [34, 35], primary laboratory animal MPCs [36–38], pluripotent stem cell (PSC) derived myocytes [39–41] and primary human MPCs [42–45] have been demonstrated, but relatively few of these engineered skeletal muscles have been shown to possess a regenerative capacity following injury [46–49].

The regenerative processes of some engineered muscle models have shown clear correlations to that of in vivo muscle, and so the utilisation of these engineered tissues in studies of regeneration is a clear opportunity to increase our understanding of skeletal muscle regenerative physiology [46, 49, 50]. Previous engineered models of regeneration have used the snake venom cardiotoxin (CTX) to induce a chemical muscle injury. CTX is widely used in animal models to produce a specific cellular model of muscle injury and has been shown to be effective in engineered tissues [49, 50]. The model presented here takes a similar approach utilising Barium chloride (BaCl_2_), which is also widely used in laboratory animals as a specific myotoxin and produces a comparable injury type [51]. BaCl_2_ was chosen as an injurious stimulus due to previous in vivo publications, its high water solubility and ready availability with low regulatory restrictions, allowing easy and reproducible in vitro application [20, 51]. In addition, the mechanism of injury following BaCl_2_ treatment is a simple cellular injury specifically removing myotubes without reducing mononuclear cell number. Although chemical insults do not mimic all of the damage, specifically extracellular matrix destruction caused by mechanical injuries, usually seen in vivo these insults produce a specific and reproducible injury phenotype to ensure accurate model development [46].

To ensure that data produced by these engineered models is as relevant as possible and that these models are exploited to their full potential, engineered muscles utilising primary human cells, with a regenerative capacity mimicking that of in vivo muscle, should be developed. To account for the heterogeneity of cells found within native muscle, primary tissue-derived MPCs and not human iPSCs present the most biomimetic option for creating a representative model of human skeletal muscle regeneration.

Here we present a robust, high-content protocol to generate functional engineered human skeletal muscles from primary human MPCs. Utilising cell population sorting and media optimisation, we present human engineered skeletal muscles which regenerate function and morphology completely following injury. These engineered muscles in addition to supporting regeneration also contain a self-renewing stem cell niche, presenting an opportunity to accurately study the biology of human skeletal muscle regeneration ex vivo.

## Results

### Remixing CD56+ and CD56− cell populations produce robust tissue engineered muscles

The sorting of non-myogenic and myogenic populations from human explant cultures allows extended culture periods within the myogenic populations without a significant loss of desmin positivity, a marker of myogenic potential (Additional file 1: Fig. S1a/b). However, the use of only myogenic cells in collagen/Matrigel® hydrogels produces engineered muscles of highly variable quality due to the apparent inability of these cells to reproducibly deform the hydrogel matrix (Additional file 1: Fig. S1c/d). However, due to the high proportion of myogenic cells in the CD56+ fraction (referred hereafter to as CD56+) constructs these engineered muscles, when successful, produce significantly more myotubes than unsorted equivalents (Additional file 1: Fig. S1 e-i).

The CD56− population contained predominantly TE7-positive cells, most likely to be interstitial fibroblasts (70.9 ± 28.7%, Additional file 1: Fig. S2), with a small percentage of myogenic cells (desmin positive; 5.5 ± 6.1%, Additional file 1: Fig. S2). The remainder of cells were identified utilising flow cytometry showing the presence of other muscle associated cell types, such as PDGFRα+ (Fibro–Adipogenic Progenitors; FAPs; 1.16 ± 1.24%), CD90+ (mesenchymal stem cell marker; 13.9 ± 13.0%), CD45+ (immune cell lineage; 2.03 ± 1.66%) and CD31+ (endothelial cell marker; 5.60 ± 4.86%). All of these cell types were present as relatively small percentages of the population when compared to the TE7 population (Additional file 1: Fig. S2).

**Fig. 1:**
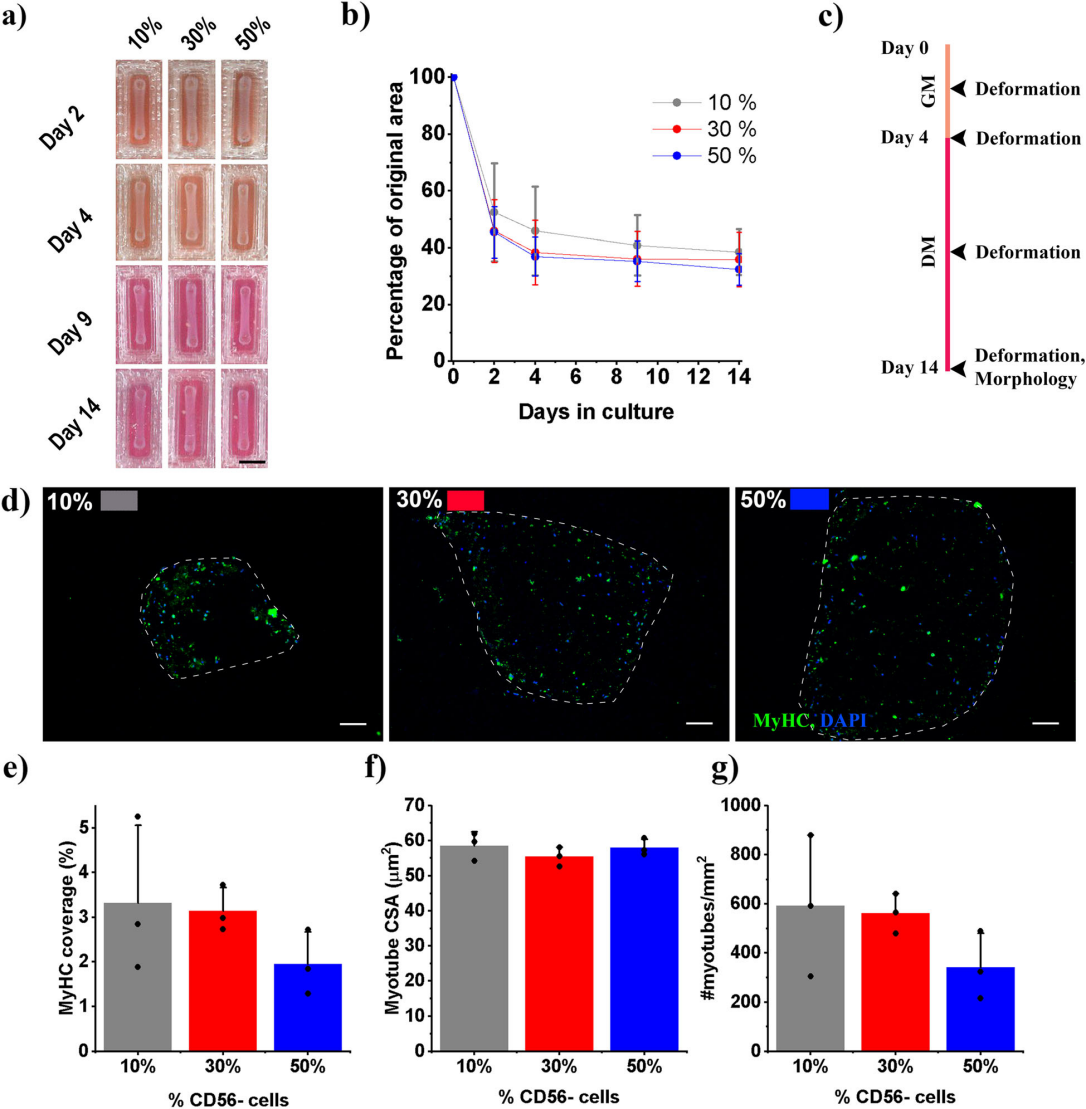
Remixing of CD56-sorted populations leads to robust engineered muscles. Throughout percentages refer to the percentage of CD56- cells remixed to create the human cell population. Black in all graphs denotes 10%, red 30% and blue 50% CD56− .**a** Photographs of engineered muscles across time showing deformation. Scale bar – 5 mm. **b** Deformation over time. **c** Experimental scheme showing time points of analysis. **d** Representative micrographs stained for MyHC – green and Nuclei (DAPI) – blue. Scale bar 100 μm. **e–g** Graphs displaying; MyHC percentage coverage, myotube cross-sectional area (CSA) and myotubes per mm^2^. All graphs display mean ± S. D, individual repeat means are displayed as points. No statistically significant comparisons were identified, *n* = 9 samples across 3 repeats

To exploit the high myogenic potential of CD56+ cells, a dose remixing experiment was undertaken to identify the lowest proportion of CD56− cells required to reproducibly deform collagen/Matrigel® constructs. The deformation of these constructs is a key feature of their development, generating tension to align developing myotubes. The CD56− cell fraction was therefore remixed with the CD56+ fraction at various ratios (10% 9:1 CD56+:CD56−, 30% 7:3 CD56+:CD56− and 50% 1:1 CD56+:CD56−) and hydrogel deformation and morphological appearance of the tissue examined. All ratios produced engineered muscles which deformed robustly, without any significant difference between conditions (Fig. 1a/b). Clear trends in morphological appearance were present across conditions with 10% CD56− constructs displaying the highest number of myotubes per square mm and the largest percentage of constructs occupied by myotubes (Fig. 1d, e, and g). A small decrease between 10 and 30% CD56− and a much larger step between 30 and 50% CD56− engineered muscles was observed indicating that increasing the CD56− fraction reduced myogenic potential (Fig. 1d, f). As the proportion of CD56− cells increased, these measures of myotube formation were reduced, although this trend was not significant. Myotube cross-sectional areas (CSA) remained unaffected by the proportion of CD56− cell included in constructs (Fig. 1f). As 10% CD56− remixing produced robust deformation and allowed the maximum inclusion of the CD56+ myogenic fraction, this remixing ratio was carried forward for all future experiments.

### Media supplementation increases morphological and functional markers of muscle maturity

**Fig. 2:**
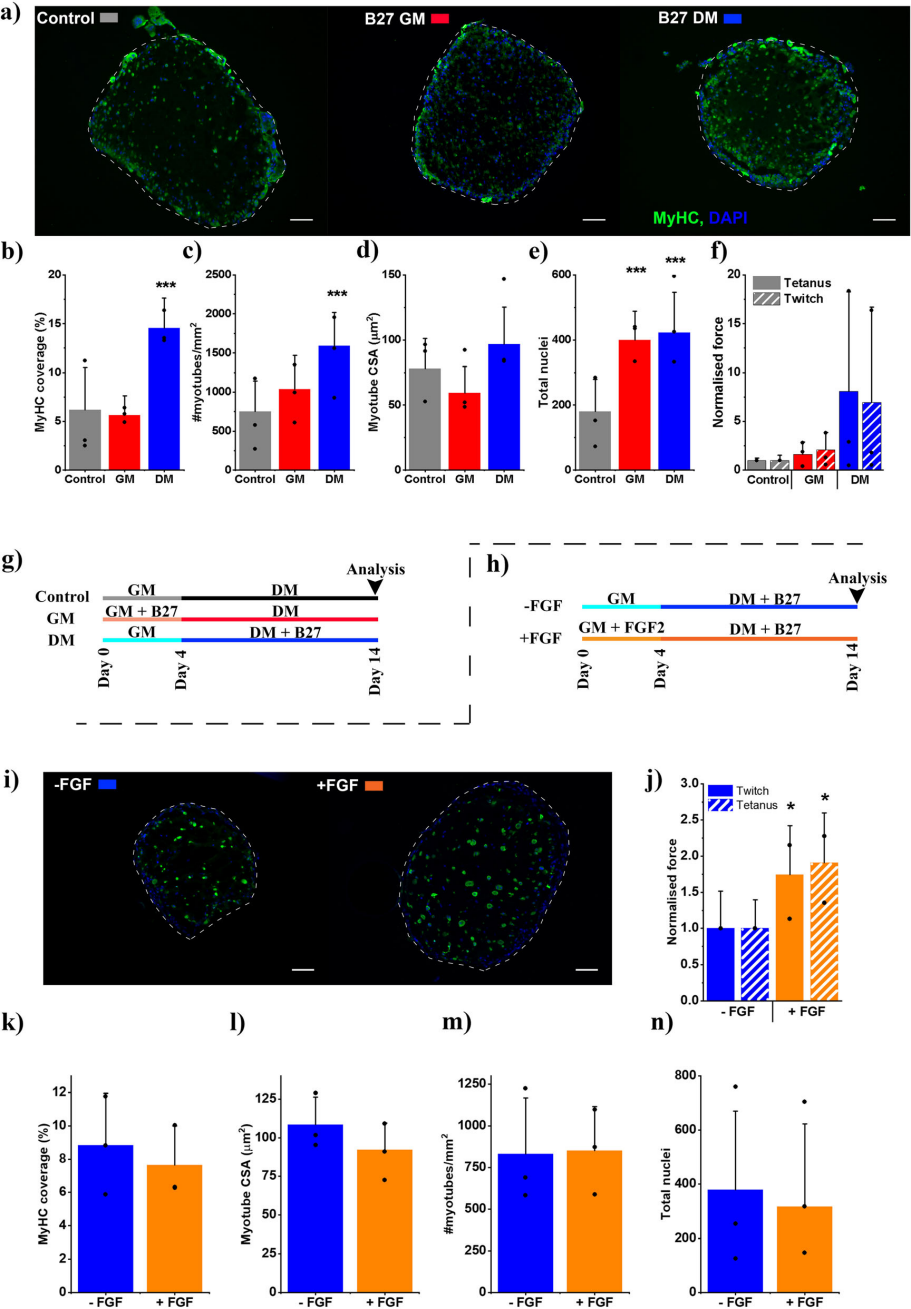
Media supplementation of remixed engineered muscles increases morphological maturity and increases functional capacity. **a** Representative micrographs stained for myosin heavy chain (MyHC) – green and Nuclei (DAPI) – blue. Scale bar 100 μm. **b–f** Graphs displaying; percentage MyHC coverage, myotubes per mm^2^, myotube cross-sectional area (CSA), nuclei per section and normalised force (normalised to control), respectively. Mean tetanus force at control 45.9μN, 0.96 kPa; twitch force 22.7μN, 0.048 kPa. All graphs display mean ± S. D. **g**, **h** Experimental schemes showing conditions coloured similarly to graphs. Scheme (**g**) applies to **a**–**f** whilst scheme (**h**) applies to **i**–**n**. **i** Representative micrographs stained for myosin heavy chain (MyHC) – green and Nuclei (DAPI) – blue. Scale bar 100 μm. **j** Normalised force (normalised to -FGF condition) mean tetanic force for -FGF condition 14.3μN, 0.10 kPa; twitch 6.2μN, 0.35 kPa. **k**–**n** Graphs displaying; Percentage MyHC coverage, myotubes per mm^2^, myotube cross-sectional area (CSA), nuclei per section respectively. All graphs display mean ± S. D, individual repeat means are displayed as dots. Statistical significance from control (**a**–**f**) and -FGF (**i**–**n**) is denoted as *p ≤ 0.05, ***p ≤ 0.001, *n* = 9 samples across 3 repeats

Engineered muscles (CD56−:CD56+, 10:90) were supplemented either in the growth phase (days 0–4) or the differentiation phase (days 4–14) of culture with 2% B-27 supplement. Supplementation with B-27 increased nuclei number approximately 2-fold irrespective of the phase in which it was added (*p* = 0.001, *p* = 0.004, Fig. 2e). However, only supplementation in the differentiation phase of culture lead to increases in total myosin heavy chain (MyHC) coverage, myotube number and myotube CSA (*p* < 0.001, *p* < 0.001, *p* = 0.004, Fig. 2a–d). No significant changes in force generation were observed in any B-27 supplementation conditions; however, supplementation in the differentiation phase of development did lead to a mean increase in tetanic force of 8-fold, although this response was highly variable between repeats.

Supplementation of GM with FGF2 at 5 ng/mL followed by 10 days in B-27 supplemented DM (+FGF, Fig. 2) was compared to unsupplemented GM followed by B-27 supplemented DM (−FGF, Fig. 2). FGF2 supplementation did not change any morphological measure significantly (Fig. 2i, k–n). However, FGF2 addition did increase force generation significantly for both tetanus (1.9-fold, *p* = 0.017) and twitch (1.74-fold, *p* = 0.044). This data allowed the selection of FGF2 supplemented GM and B-27 supplemented DM as suitable medias for the culture of 10% CD56− engineered human skeletal muscles.

### Human engineered muscles contain multinucleated myotubes, Pax7+ nuclei and laminin organisation

**Fig. 3:**
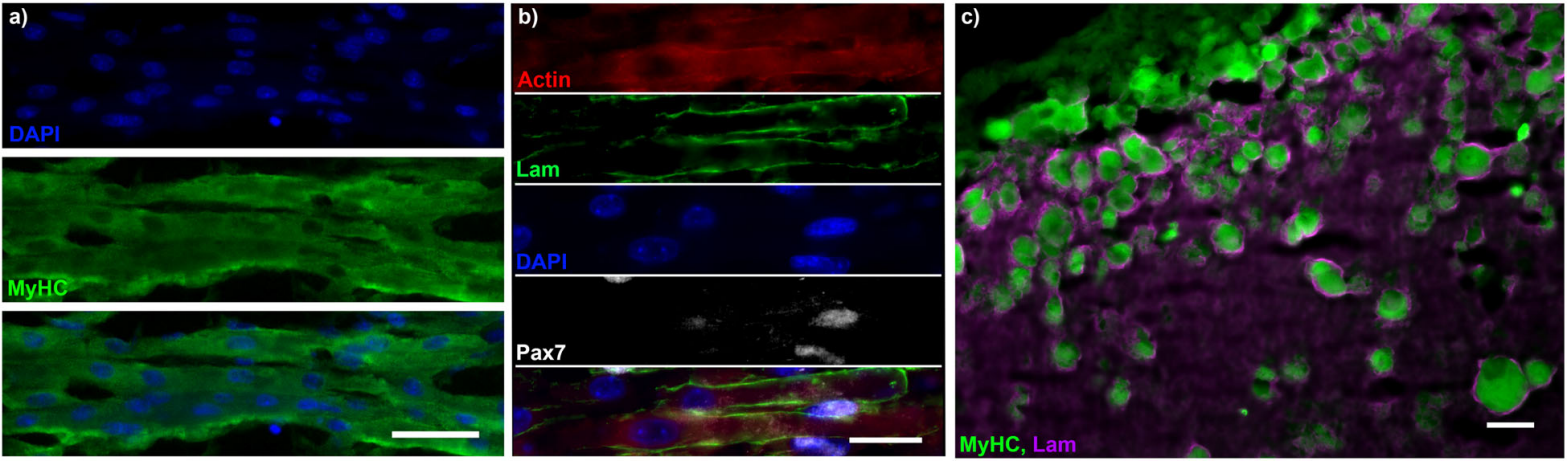
Human engineered muscles display laminin organisation and Pax7+ nuclei. **a** Micrograph of longitudinal cryosection of engineered muscle. Stained for nuclei (DAPI, blue) and myotubes (MyHC, green). **b** Micrograph of longitudinal cryosection of engineered muscle. Stained for Actin (red), Laminin (green), DNA (DAPI, blue) and Pax7 (white). **c** Cross-section of engineered muscle imaged on the extreme periphery of the section. MyhC (green) and Laminin (magenta). All scale bars represent 25 μm

To identify how similar engineered skeletal muscle was in its matrix and cellular organisation to somatic muscle, features of in vivo muscle morphology were examined. Longitudinal staining for MyHC confirmed that structures positive for MyHC were multinucleated myotubes (Fig. 3a). Laminin staining of cross-sections showed clear and distinct concentrations of laminin staining surrounding virtually all myotubes within engineered muscles, closely reminiscent of the basement membrane organisation of in vivo muscle (Fig. 3b/c). However, another basement membrane component and component of Matrigel®, collagen IV, did not show a similar organisation, suggesting this enrichment is specific for laminin and not a general compression of the matrix (Additional file 1: Fig. S3). Longitudinal analysis showed the presence of Pax7+ nuclei, with a small proportion of these associated with a laminin rich area of matrix or the plasma membrane of myotubes (Fig. 3b).

### Engineered skeletal muscles are capable of functional and morphological regeneration following injury

**Fig. 4:**
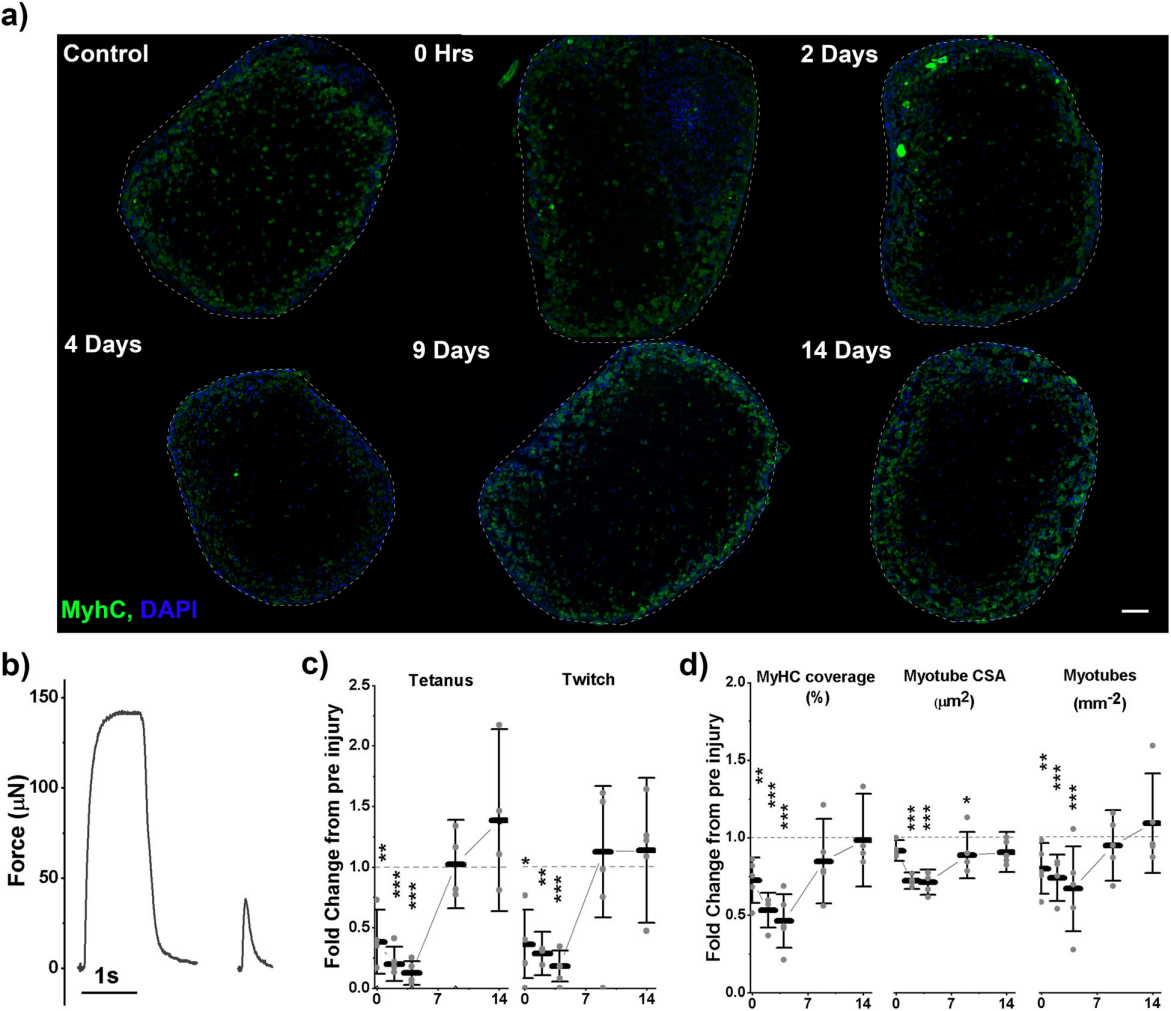
Human engineered muscles regenerate functionally and morphologically following injury. **a** Representative micrographs of engineered muscle cross-sections. Stained for MyHC (green) and Nuclei (DAPI, blue). Scale bar represents 100 μm. **b** Representative force traces used for tetanus and twitch force measurements. **c** Normalised force measurements across recovery. Means at control; tetanus – 101.6 μN, 0.19 kPa; twitch – 36.6μN, 0.072 kPa. **d** Normalised morphological measures across recovery. Means at control; percentage MyHC coverage – 13.0%, myotube cross-sectional area (CSA) – 132.6 μm^2^, myotubes per mm^2^ –971.6. **b**–**d** All graphs display control normalised means ± S. D, individual repeat means are displayed as points. Dashed line represents level at control, normalised to 1, on all graphs. Statistical significance from control is denoted as *p ≤ 0.05, **p ≤ 0.01 and ***p ≤ 0.001, *n* = 15 samples across 5 repeats

To examine the regenerative capacity, and so the function of the Pax7 niche, engineered muscles were exposed to an injurious stimulus in the form of BaCl_2_ exposure. Injury caused an initial reduction of 27.5% in MyHC positive coverage (*p* = 0.006) and this reduction in coverage became more pronounced up to 4 days post injury (54.7%, *p* < 0.001, Fig. 4a, d). This reduction was caused predominantly by a loss of myotubes (myotubes per square mm), from 972 mm^−2^ pre-injury to 789 mm^−2^ immediately post injury (*p* = 0.005, Fig. 4a, d) further reducing to 638 mm^−2^ (*p* = 0.001) 4 days post injury. This reduction was also accompanied by moderate atrophy of myotubes, with CSA reducing 9% following injury, although not significantly. This atrophy increased to a maximum of 29% (38.9 μm^2^, *p* < 0.001) at 4 days post injury. Across all measures of myotube size and density, the largest reduction was seen between control and immediately post injury (6 h BaCl_2_ incubation); however, these measures continued to decline across the first 4 days of regeneration. At 9 days post injury, MyHC coverage had recovered to 85% of uninjured levels and was no longer significantly reduced. In addition, myotubes per square mm had returned to 94% of uninjured controls, suggesting that myotube CSA was still depressed. Indeed, myotube CSA at day 9 post injury was significantly reduced, with an average CSA of 115 μm^2^ compared to 133 μm^2^ at control (*p* = 0.05, Fig. 4a, d). Following the full 14 days of regeneration, all measures of myotube density and size had returned to control levels showing complete morphological regeneration of the tissue.

With morphological change, an accompanying reduction in functional output is expected. Immediately following injury, both tetanic and twitch force were reduced by an average of 62% compared to control (*p* = 0.003, *p* = 0.024, Fig. 4c). Both measures of function remained significantly depressed at 2 days and 4 days post injury when compared to control. At day 9 post injury, both twitch and tetanic force were recovered to control levels and remained comparable to control at the end of regeneration at 14 days post injury (Fig. 4c).

### Dynamics of myogenic and non-myogenic cell populations during regeneration

**Fig. 5:**
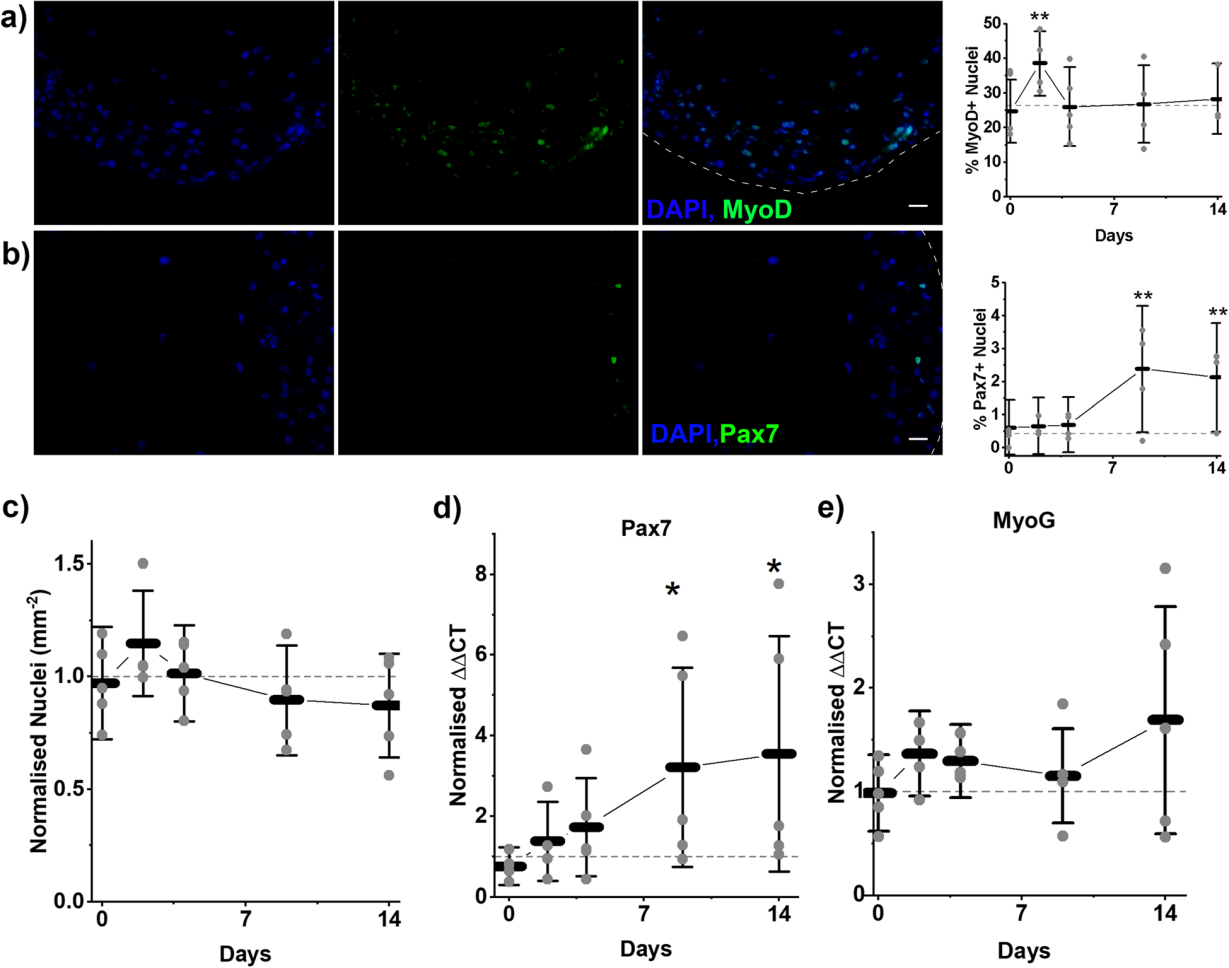
Dynamics of cell populations following injury. **a** Representative images showing staining left to right, Nuclei (DAPI, blue), MyoD (green) and overlay image. Scale bar represents 25 μm. Graph displays percentage of MyoD+ nuclei across recovery. **b** Representative images showing staining left to right, Nuclei (DAPI, blue), Pax7 (green) and overlay image. Scale bar represents 25 μm. Graph displays percentage of Pax7+ nuclei across recovery. **c** Normalised nuclei per mm^2^ across recovery, mean value at control – 1471 mm^− 2^. **d** RT-PCR data displayed as ΔΔCT values for myogenic genes Pax7 and MyoG across recovery. **a**–**e** All graphs display means ± S. D, individual repeat means are displayed as points. Dashed line represents level at control on all graphs. Statistical significance from control is denoted as *p ≤ 0.05, **p ≤ 0.01 and ***p ≤ 0.001, *n* = 15 samples across 5 repeats

Nuclei per square mm was recorded to ensure that engineered muscles remained viable throughout recovery, and no significant variation in this measure was observed (Fig. 5c). However, an increase of 15% was observed 2 days post injury (*p* > 0.05), although this was resolved by 4 days post injury. To examine if different populations of cells within the overall population were expanding in relation to nuclear markers of the myogenic lineage, Pax7 and MyoD were stained for and expressed as a percentage of total nuclei. MyoD, which marks proliferative myoblasts committed to the myogenic lineage, showed no change immediately following injury. However, a significant increase from 26.4% at control to 38.6% was observed after 2 days of regeneration (*p* = 0.005, Fig. 5a). This expanded MyoD population was completely collapsed following a further 2 days, at 4 days post injury, with the percentage of MyoD-positive nuclei returning to 26.0%. Through the remainder of regeneration, the percentage of MyoD-positive nuclei remained comparable to control. Pax7 a marker of satellite cells in vivo was found to be initially rare, making up only 0.42% of total nuclei at control, and no significant changes in Pax7-positive nuclei percentage was observed in the first 4 days following injury. However, following 9 days of regeneration Pax7+ nuclei comprised 2.4% of total nuclei a significant increase (*p* = 0.002, Fig. 5b) which persisted until 14 days post injury where Pax7-positive nuclei comprised 2.1% of total nuclei (*p* = 0.004, Fig. 5b).

Examination of Pax7 expression by RT-PCR showed a similar trend as Pax7 staining. Pax7 mRNA expression increased throughout recovery with 3.2- and 3.5-fold increases at 9 and 14 days post injury being significant (*p* = 0.02, *p* = 0.015). Although the 3-fold changes observed by RT-PCR are smaller than the approximate 6-fold change observed in staining, the trends appear to be consistent, with increased Pax7+ nuclei appearing through the final 10 days of regeneration (Fig. 5d). Myogenin (MyoG) expression was also analysed, as a later myogenic marker than MyoD, it would be expected to follow a similar trend but temporally delayed. The trend of MyoG expression is broadly similar to MyoD-positive nuclei with a rise following 2 days of regeneration and partially resolving at day 9 post injury. An increase at 14 days post injury is observed although this is not statistically significant (Fig. 5d).

The non-myogenic transcription factors Runx2 and Pparg were also examined to illuminate the dynamics of potentially non-myogenic cells which in vivo can drive the formation of non-regenerative defects such as increased interstitial fat and heterotopic bone. Runx2 mRNA showed no statistically significant deviation from control levels, although an increase was observed at all time points across the first 9 days of regeneration (Additional file 1: Fig. S4). Pparg mRNA was significantly increased throughout regeneration, with the exception of immediately post injury. At 2 and 4 days post injury, Pparg mRNA expression was elevated 1.88- and 1.89-fold, respectively (*p* < 0.001, *p* < 0.001). This expression was reduced to 1.37-fold at 9 days post injury (*p* < 0.001) before elevating again to 2.16-fold (*p* < 0.001, Additional file 1: Fig. S4) after 14 days of regeneration. The upregulation of Pparg suggests a level of non-myogenic repair occurring in response to injury.

## Discussion

Here we have demonstrated, by the exploitation of well-established tissue engineering approaches, a protocol which allows the generation of engineered human muscle with the capacity to regenerate function following injury. The tissues generated are functional, contain the mixed cell populations represented in muscle and demonstrate myogenic population dynamics reminiscent of in vivo tissue. Although previous work has shown that engineered muscles can regenerate following injury [46–49], no previous work has demonstrated functional recovery in human engineered muscles [52]. The injury sustained following BaCl_2_ treatment is robust, leading to significant loss of myotubes and function. As with in vivo skeletal muscle wound healing and previous primary tissue engineered muscles, injury is followed by a period (in this model 4 days) of impaired function and reduced myotube number [48, 49, 51]. This contrasts with our previous cell line based 3D models, which isolates the response of committed myogenic cells, and shows a very rapid recovery of function with no prolonged post injury period [46]. Following this initial period without myotube formation, a complete recovery of myotube number and function was observed in line with similar injury types in animal models and previous engineered tissues [46, 47, 49, 51]. This demonstrates the regenerative capacity of human skeletal muscle ex vivo and positions this model as a useful tool in examining the underlying biology of skeletal muscle regeneration and repair.

Initially, we demonstrate that the remixing of CD56− and CD56+ cells is required to create robust engineered muscles in line with our previous work [45, 53]. Other models have shown that it is possible to generate CD56+ only engineered tissue, but this work utilises a fibrin-based hydrogel and an FGF2 supplemented myogenic growth media and these differences may explain the contrasting requirement for CD56− cells [54]. This protocol demonstrates that only a very low proportion of non-myogenic cells are required to drive hydrogel deformation, allowing a high percentage of myogenic cells to be exploited (Fig. 1). MidiMACS sorting is unlikely to be absolutely efficient in producing a population of homogeneous cells on the basis of CD56 expression; however, the desmin positivity values in excess of 90% observed here suggest a highly efficient sorting yield. In addition to increasing myogenic potential, sorting and remixing reduces donor to donor variation in desmin positivity, a common observation with hMPCs, reducing some of the donor variability and likely increasing experimental power. Finally, sorting allows separate expansion of myogenic and non-myogenic populations and therefore extends the usability of hMPCs. hMPCs unsorted rarely retain sufficient desmin positivity for engineered tissue after 6 passages, and therefore a standard microbiopsy sample would yield approximately 100 × 50 μL engineered muscles. However, sorting cells allows further expansion (up to at least 9 passages, which could potentially be extended) and a projected 20-fold increase in MPC yield allowing 2000 engineered muscles to be generated per biopsy sample. Taken together, these advantages suggest clear rational for exploiting CD56 sorting for hMPC tissue engineering and solves one of the key limitations of using hMPCs.

Remixing of separate populations produces a method to increase cell yield and reproducibility of engineered human skeletal muscle by allowing the expansion of competing populations in isolation. A remixing ratio of 10:90 (−ve:+ve) was selected as this ratio allowed the maximum percentage of myogenic cells to be incorporated into a construct which reproducibly remodelled to generate tension between the anchor pins. We did not examine more in-depth markers of remodelling, such as matrix metalloproteases (MMPs) or their tissue inhibitors (TIMPs), which may be of interest in future work to refine this model. In addition to remodelling the matrix, the CD56− fraction contains a range of other cell types found within the muscle (Additional file 1: Fig. S3) which have been shown in vivo to play important roles in muscle regeneration. This remixing is therefore also key to preserving the biological validity and utility of hMPCs. Identified through flow cytometry PDGFRα+ cells (FAPs), CD90+ (MSCs), CD45+ cells (immune lineage) and CD31+ (endothelial cells) were all identified alongside TE7+ cells (interstitial fibroblasts). The sum of the population fractions, although calculated across two methods, approximates at 99% of the total population suggesting that the predominant lineages within this population are accounted for, although not confirmed beyond surface marker expression. All of these cell types have suggested roles within the regenerative process and their inclusion may underpin some of the regenerative capacity of this model and at the very least provide an opportunity to examine in vitro how these populations behave following muscle insult.

However, even utilising CD56 sorting, without media supplementation engineered muscles of this type have relatively low numbers of myotubes and produce functional output close to the limit of detection reducing the usefulness of this key measure. To further improve the quality of engineered tissues growth factor supplements (FGF2) and commercial supplement cocktails (B-27) were used. B-27 supplement is widely used in primary neuronal and stem cell cultures [55, 56]. The supplement contains a broad range of components aimed at promoting redox balance, increasing metabolic flexibility, providing trace nutrients and driving cell growth with supplemental growth factors (Insulin) and steroids (triiodothyronine (T3), corticosterone and progesterone). The roles of these components are broad with protection from redox stress and provision of trace nutrients likely to promote cell survival and lead to increased cell numbers [57]. Growth factors and steroid hormones are more likely to have cell type-specific effects, with insulin having been shown in myoblasts to increase fusion and the expression of myogenic genes, as well as driving proliferation [58, 59]. Thyroid hormones have well-established roles in regulating skeletal muscle growth and differentiation, with hypothyroidism leading to reduced muscle mass [60]. At a molecular level, the action of T3 on the expression of myogenic genes, MyoD and Myogenin, has been clearly established suggesting T3 will drive myogenesis in engineered muscles [61, 62]. The effects of progesterone on skeletal muscle remain relatively unexamined, with the expression of progesterone receptors yet to be confirmed in myoblasts [63]. Finally, corticosterone, which has only a limited role in humans with its ortholog cortisol being the predominant corticosteroid, drives skeletal muscle atrophy and inhibits insulin signalling in vivo and may perform a similar function in culture [64]. The paucity of data makes the action of progesterone, and the apparent adversarial effects of insulin vs corticosterone, makes it difficult to unpick precisely the role of each component in B-27 and future work may be required to better understand the mechanisms by which the supplement drives myogenesis. Previous work using B-27 with skeletal muscle myoblasts has been shown to promote myoblast survival, but not differentiation in primary rat MPCs [65] whilst as a serum replacement for iPSCs B-27 promotes myogenesis [56]. In the first case, B-27 was included throughout culture and may better compare with the addition of B-27 to the growth phase in this work where increases in cell number were observed but no increase in myogenesis. Whereas in the work by Jiwlawat et al., the addition of B-27 occurs to support terminal differentiation, which is similar to the addition of B-27 in the differentiation phase in this work.

The growth factor FGF2 is known to promote myoblast proliferation, matrix remodelling and attachment and is widely used in MPC culture [45, 66] whilst also inhibiting differentiation [67, 68] and so was only used in the growth phase of engineered muscles. A doubling in force production shows an effect of supplementation, but this is not driven by an increase in nuclei at maturity. It is possible proliferation may happen earlier in FGF2 supplemented muscles or that there is a priming effect of FGF2 which leads to increased maturity and so force production. The data presented here does not allow this to be examined further. Together, this data demonstrates that supplementation effectively improves the quality of engineered muscle (MyHC coverage and force production) produced from hMPCs and although this may not be the absolute optimal supplementation protocol presents a viable solution to produce functional human engineered muscle (Fig. 2).

Although myotube CSA and MyHC coverage are low compared to somatic human muscle [69], morphological examination of engineered tissues reveals an organised basement membrane, shown by laminin rings surrounding muscle fibres and the presence of Pax7-positive nuclei (Fig. 3). However, the niche present in native muscle contains approximately 5–8% of Pax7+ nuclei, substantially greater (10/15-fold) than observed in control engineered tissues [70, 71]. This may be explained by a lack of developmental cues such as the exercise/injury seen in vivo which activate satellite cells and cause proliferation [72]. These Pax7+ cells, however rare, are a key feature of skeletal muscle, underpinning the regenerative capacity of in vivo skeletal muscle and supporting tissue growth and turnover, and therefore should be present in models of engineered skeletal muscle [2, 5, 73].

To examine if these engineered tissues follow similar patterns of regeneration as native skeletal muscle, myogenic and non-myogenic markers were examined through protein and mRNA expression. During the initial 2 days following injury, a proliferative response of MyoD+ myoblasts was observed, before a return to pre-injury levels, a response consistent with in vivo data. However, no increase in Pax7+ nuclei was observed during this period as would be expected from in vivo data [7]. This lack of Pax7 proliferation could be due to a lack of activation of these cells following injury, with regeneration driven instead by unfused MyoD+ MPCs, or due to the very low percentages of Pax7+ nuclei present making any changes difficult to detect. Distinguishing between these possibilities is difficult. However, the increases in mRNA expression of Pax7 and Pax7+ nuclei later in regeneration suggest a capacity to expand this cell population, increase the proportion of these cells potentially mimicking the self-renewing capacity of satellite cells in vivo [74, 75]. It cannot however be absolutely confirmed, without an equivalent of a contralateral control, which is not included, that the increased prevalence of Pax7+ nuclei observed could be due to repeated growth and differentiation phases, and not solely driven by the injury response. Indeed, the post injury proportion of Pax7+ nuclei (2.1%) is more closely aligned to in vivo proportions (4–7%) than the pre-injury (0.4%), suggesting that an injury stimulus may be required to trigger population expansion. We have however not presented any direct evidence here that the Pax7+ cells present in this model support regeneration directly. To achieve this, lineage tracing experiments with persistent Pax7-dependent markers would be required. Instead, it is possible, as suggested above, that unfused nuclei within the model, often referred to as reserve cells, support regeneration following injury and not the Pax7+ cells. As the fusion index of control engineered muscles is estimated at approx. 40% there are significant numbers of unfused Pax7- nuclei which could support regeneration. It is not possible to accurately suggest the relative contribution of these two populations without robust tracing experiments. The data presented does show that engineered human tissues can mimic some of the key events of regeneration, including the expansion of MPCs and the expansion of the Pax7+ cell population alongside the recovery of function and myotubes making these engineered tissues an attractive model for understanding skeletal muscle regenerative physiology (Figs. 4 and 5).

Non-myogenic markers Pparg and Runx2 drive non-regenerative repair defects in vivo [76, 77]. Pparg showed an upregulation across regeneration, although this was lower in magnitude than non-regenerative expression in vivo [77], and may be due to increased myotube or MPC expression and not indicative of adipogenic differentiation [78]. Runx2 does not show significant upregulation although some variation from control is observed (Additional file 1: Fig. S4). As remixed engineered muscles contain a range of cell types obtained from skeletal muscle, these expression patterns may represent the expansion, or increased activity of non-regenerative cells types, which should allow future work to examine how these populations progress to develop non-regenerative defects and how they may be manipulated to improve clinical outcomes.

Currently, three published models show a regenerative skeletal muscle [47–49, 52]. Of these, two are collagen-based engineered tissues, whilst the third is a fibrin-based system. All systems have examined injury through the application of cardiotoxin (CTX), with one system also utilising a crush injury. The most recent, from Rajabian et al., shows the regeneration of myotubes of human engineered tissues in both a collagen- and fibrin-based system, although does not examine engineered tissue function. The collagen-based system regenerates myotubes following 5 days of regeneration, but no further analysis is presented to compare to the data presented here, although further analysis is undertaken in a fibrin-based model. The remaining collagen-based system, Tiburcy et al., utilises primary rat MPCs and shows a m-cadherin-lined SC niche and has a regenerative capacity similar to the model presented here, with the ability to regenerate force and morphology following an injury which completely ablates the ability of the tissue to regenerate force. In addition, this model shows a comparable response with regards to Pax7+ proliferation, with an increase across time following injury rather than a brief and temporally confined increase in Pax7+ nuclei. This is in contrast with fibrin systems containing both human and rat based MPCs which display a Pax7 proliferative wave and subsequent resolution. Interestingly, with a crush injury as opposed to chemical insult, Tiburcy et al. demonstrate that collagen-based engineered muscles display this peak in Pax7 and resulting resolution rather than in progressively increasing population. Fibrin-based systems appear to have a more limited regenerative capacity in response to significant functional injury. Both this model and that of Tiburcy et al. show complete ablation of force following injury and complete recovery; however, Juhas et al. see a limit of 50% functional reduction before regeneration no longer occurs, even in the presence of regeneration supporting macrophages. In summary, although this model remains the only model to show the functional regeneration of a human engineered tissue, it broadly shares similar characteristics with other published models. Interestingly, the closest comparison can be made with the collagen/Matrigel® based system of Tiburcy et al. which utilises rat MPCs bringing into focus the key role of matrix composition in these engineered tissues.

## Conclusions

The model presented here provides a platform to generate large numbers of tissue engineered muscles from a single microbiopsy. Utilising CD56 sorting and media supplementation this protocol is robust and allows researchers, in combination with the open source mould system, to rapidly generate human skeletal muscle tissues within their laboratory [79]. The demonstration that these tissues regenerate following chemical insult allows the study of human skeletal muscle regeneration, including cell population dynamics across time, to be undertaken without the need to invasively sample patients repeatedly. In addition, the flexibility of the system allows for future work to build complexity, such as through the addition of immune cells to simulate an inflammatory response [48] or mechanical/electrical stimulation to capture the effects of post-injury exercise [34, 44]. As the complexity and maturity of these models develop, they will present an opportunity to test putative clinical interventions in a high/medium throughput manner on human tissue, adding a novel tool to the preclinical testing tool box to help improve lead screening and ultimately improve healthcare for patients.

## Methods

### Isolation and culture of hMPCs from skeletal muscle biopsies

Participants were recruited according to Loughborough University ethical and consent guidelines (Ethics no. R18-P098), with anonymised participant characteristics presented in Additional file 1: Table S1. Biopsies were collected by microbiopsy method from the vastus lateralis [80]*.* All collected tissue was minced finely, and connective tissue removed. Minced tissue was then plated out and cells isolated by explant culture [43, 55]. Once collected cells were expanded to passage 3 (p3) in gelatin-coated (0.2% v/v) culture flasks. At p3 cells were sorted for the presence of the myogenic cell surface marker CD56 [81], using a MidiMACS™ system (Miltenyi Biotech, DE). Further expansion of the separate populations was then undertaken, CD56+ cells in Corning® Matrigel® basement membrane matrix-coated (1 mg/mL, Fisher Scientific, UK) flasks and CD56− cells in gelatin solution (Sigma-Aldrich, UK)-coated flasks. At p5 cells were cryopreserved or further expanded and used between p7 and p9. Throughout explant and expansion cells were maintained in growth media (GM – 79% high glucose Dulbecco’s modified Eagle’s medium (DMEM, Sigma, UK), 20% fetal bovine serum (FBS, PanBiotech, UK) and 1% penicillin/streptomycin (P/S, Fisher, UK)). For the culture of minced tissue, 1% Amphotericin B (Sigma, UK) was added to standard GM. At no point were flask cultures allowed to exceed 65% confluence and were split at a ratio of 1:3 at passage.

### Generation of tissue engineered muscles

Engineered muscles were made as described previously [43, 46]. Briefly, 65% v/v acidified type I rat tail collagen (2.035 mg/mL, First link, UK) and 10% v/v of 10× minimal essential medium (MEM, Sigma) were mixed and neutralised. This was followed by the addition of 20% v/v Matrigel® and 5% v/v GM containing hMPCs at a final density of 4 × 10^6^ cells/mL and in a ratio of 9:1 CD56+:CD56− unless otherwise stated. The final solution was transferred to pre-sterilised biocompatible polylactic acid (PLA) FDM printed removable box 50 μL inserts [82] to set for 10–15 min at 37 °C. All moulds used in this manuscript are freely available to download at the following URL: https://figshare.com/projects/3D_Printed_Tissue_Engineering_Scaffolds/36494. Engineered skeletal muscles were maintained in GM with 5 ng/mL FGF-2 (Peprotech, USA) for 4 days, changed every 48 h. Following 4 days media was changed to differentiation media (DM – 97% DMEM, 2% horse serum and 1% P/S) supplemented with Gibco™ B-27™ Supplement (50×, 1:50, Sigma) for a further 10 days.

### Barium chloride injury and regeneration

Once engineered muscles had reached maturity (14 days), as defined above, they were exposed to chemical injury by BaCl_2_. Prior to inducing injury, fresh DM was added to all conditions. Precisely 50 μL/mL of 12% w/v BaCl_2_ solution was then added to the medium for injury culture conditions, followed by a 6 h incubation to induce injury. Addition of BaCl_2_ to cell culture media may produce a white precipitate which has the potential to obscure immunohistochemical analysis. For applications not utilising sectioning techniques for imaging, non-phosphate/sulphate buffers can be considered to prevent precipitate formation [83]. Following injury, cultures were washed once with phosphate-buffered saline (PBS) to remove residual BaCl_2_ containing media. Control (no injury) and 0 h (0 h) time points were collected at the end of injury incubation. Additional time points at 2, 4, 9 and 14 days post injury were collected for all measures to examine the regenerative response across time. For the first 4 days of regeneration engineered muscles were maintained in GM with FGF2, and the remaining 10 days DM with B-27.

### Tissue fixation, sectioning and staining

Engineered muscles were fixed in 3.75% formaldehyde solution overnight at 4 °C and then stored in PBS. Prior to cryosectioning, engineered muscles were stored in 20% sucrose solution w/v for 24 h at 4 °C to reduce water content and then were frozen under isopentane in liquid nitrogen. Sections were then prepared using standard cryotomy methodology. Cross-sections for MyHC staining were prepared at 12 μm, whilst Pax7 and MyoD staining used 4 μm sections. Longitudinal sections were prepared at 10 μm.

Sections were incubated overnight at 4 °C with primary antibodies and 1 h with secondary antibodies (Fisher, Goat anti-mouse/rabbit 488/647, 1:500). For Pax7 (deposited to the DSHB by Kawakami, A., US, 1:125) and MyoD (Santa Cruz Biotechnology, US, sc-377,460, 1:200), antigen rescue at 75 °C in citrate buffer (pH 6) for 20 min was performed before the addition of primary antibody. For Laminin (Abcam, ab11575,1:200) and MyHC (deposited to the DSHB by Fischman, D.A., MF-20, 1:200), antigen rescue was not required. DAPI (Fisher, 1:1000) was used to stain nuclei.

Images were collected on a Leica DM2500 microscope using Leica Application Suite X software. Fiji 1.52e [84] was used for image analysis, and an in house macro performed automated myotube and nuclei analysis. Pax7- and MyoD-positive nuclei analysis was performed manually. Five random images per repeat, per measure were taken and analysed to generate the presented data.

### RNA extraction and real-time polymerase chain reaction (RT-PCR)

Engineered muscles were snap frozen upon collection and TRIReagent® extraction was augmented by mechanical disruption of constructs in a TissueLyser II (Qiagen, UK) for 5 min at 20 Hz. Following disruption, RNA extraction was carried out using chloroform extraction, according to the manufacturer’s instructions (TRIReagent®, Sigma). RNA concentration and purity were obtained by UV-Vis spectroscopy (Nanodrop™ 2000, Fisher).

All primers (Additional File 1: Table S2) were validated for 5 ng of RNA per 10 μL RT-PCR reaction. RT-PCR amplifications were carried out using Power SYBR Green RNA-to-CT 1 step kit (Qiagen, UK) on a 384 well ViiA Real-Time PCR System (Applied Bio-systems, Life Technologies, ThermoFisher, USA) and analysed using ViiA 7RUO Software. RT-PCR procedure was 50 °C, 10 min (for cDNA synthesis); 95 °C, 5 min (reverse transcriptase inactivation); and followed by 40 cycles of 95 °C, 10 s (denaturation); 60 °C, 30 s (annealing/extension). Melt analysis was then carried out using standard ViiA protocol. Relative gene expressions were calculated using the comparative CT (ΔΔCT) method giving normalised expression ratios [85]. RPIIβ was the designated housekeeping gene in all RT-PCR assays and sample controls for each primer set were included on every plate.

### Measurement of engineered muscle function

Electric field stimulation was used in order to assess the functional capacity (force generation) of tissue engineered constructs. Constructs were washed twice in PBS, and one end of the construct removed from the supporting mould pin. The free end of the construct was then attached to the force transducer (403A Aurora force transducer, Aurora Scientific, CA) using the eyelet present in the construct. The construct was positioned to ensure its length was equal to that before removal from the pin and covered (3 mL) with Krebs-Ringer-HEPES buffer solution (KRH; 10 mM HEPES, 138 mM NaCl, 4.7 mM KCl, 1.25 mM CaCl_2,_ 1.25 mM MgSO_4_, 5 mM glucose, 0.05% bovine serum albumin in dH_2_0, Sigma, UK). Aluminium wire electrodes, separated by 10 mm, were positioned parallel either side of the construct to allow for electric field stimulation. Impulses were generated using LabVIEW software (National Instruments, UK) connected to a custom-built amplifier. Maximal twitch force was determined using a single 3.6 V/mm, 1 ms impulse and maximal tetanic force was measured using a 1 s pulse train at 100 Hz at 3.6 V/mm, generated using LabVIEW 2012 software (National Instruments). Twitch and tetanus data were derived from 3 contractions per construct, and a minimum of 2 constructs per time point per biological repeat. Data was acquired using a Powerlab system (ver. 8/35) and associated software (Labchart 8, AD Instruments, UK). Force is presented as both absolute force (μN) and specific force relative to construct cross-sectional area (kPa) for comparison. Specific force was calculated using average absolute force values normalised with average associated cryosection CSA.

### Flow cytometry

CD56− cells were resuscitated from liquid nitrogen, at P6, and incubated in GM for 30 min at 37 °C, 5% CO_2_. Cells were then filtered using MACS pre-separation filters to remove potential cell clumps. Cells were then washed once in fluorescence associated cell sorting buffer (FACS; 1% BSA, 0.2 mM EDTA and 0.1% sodium azide in PBS) and resuspended in 200 μL FACS buffer, containing the appropriate antibodies (BD bioscience; PDGFRα, 556002, 1:10; CD90, 555595, 1:100; CD45, 555485, 1:10; CD31, 746116: 1:100), at a concentration of 1 × 10^6^ cells/mL and incubated for 30 min on ice. Cells were then washed with FACS and resuspended at 0.5 × 10^6^ cells/mL. Flow data acquisition was then undertaken using a BD Accuri C6 flow cytometer, at fast flow rate. To ensure accurate gating of positive populations, fluorescence minus one (FMO) controls were used to set gates. Due to the low percentages of positive cells fluorescence compensation was carried out using antibody binding compensation beads (BD, 552843). Analysis of flow data was undertaken in BD C6 software and representative gating patterns are shown in Additional file 1: Fig. S2.

### Experimental repeats

For all injury experiments (Figs. 4 and 5), 5 repeats across 3 donors were performed with each repeat yielding a minimum of 3 engineered muscles per analysis type, values from each individual engineered muscle were used for statistical analysis. For cell composition and media supplementation (Figs. 1 and 2), 2 repeats across 2 donors were performed with 3 engineered muscles per analysis technique. A total of 5 donors were used for the entirety of the experimental work.

### Statistical analysis

Statistical analysis was undertaken in IBM SPSS 23. Data was subjected to tests of normality (Shapiro-Wilk) and homogeneity of variance (Levene’s test). Where parametric assumptions were met, an ANOVA test was used to identify significant interactions. Where significant interactions were observed, Bonferroni post hoc analyses were used to analyse differences between specific time-points or groups. Non-parametric Kruskal-Wallis analysis was undertaken where data violated parametric assumptions. Mann-Whitney (*U*) tests were then used, with a Bonferonni correction, to identify the differences between groups. Comparisons across time were made between control and the time point of interested and quoted *p* values refer to this comparison. All data are reported as mean ± standard deviation (SD). Significance was assumed at *p* ≤ 0.05 and denoted on graphs with asterisks at indicated levels of significance. Without exception, an asterisk above a bar or point indicates that the mean of the indicated condition deviates significantly from the associated control.

## Supplementary information

**Additional file 1: Table S1.** Individual donor characteristics. **Table S2.** Primer sequence table. **Figure S1.** CD56 enrichment improves desmin positivity and morphological appearance of tissue engineered constructs. **Figure S2.** Characterisation of CD56- populations. **Table S3.** Table of donor sorting efficiencies and yields. **Figure S3.** Col IV and Laminin localisation in engineered skeletal muscles. **Figure S4.** Expression of Runx2 and Pparg across recovery.

## Data Availability

All data generated or analysed during this study are included in this published article and its supplementary information files.

## Abbreviations

GM: Growth medium
DM: Differentiation medium
ECM: Extracellular matrix
MPC: Myogenic precursor cell
hMPC: Human myogenic precursor cell
MyHC: Myosin heavy chain
SC: Satellite cell
VML: Volumetric muscle loss
FAP: Fibro/adipogenic cell
3D: Three dimensional
PSC: Pluripotent stem cell
DMEM: Dulbecco’s modified Eagle’s medium
FBS: Fetal bovine serum
P/S: Penicillin/Streptomycin
MEM: Minimal essential medium
PBS: Phosphate buffered saline
KRH: Krebs-Ringer-HEPES
BaCl_2_: Barium chloride
