# Supplementary material for: Bioengineered human skeletal muscle capable of functional regeneration
Source: BMC Biol. 2020 Oct 20;18:145. doi: 10.1186/s12915-020-00884-3 (PMC7576716; doi:10.1186/s12915-020-00884-3)
Supplement: Supplementary file 1 — Additional file 1: Table S1. Individual donor characteristics. Table S2. Primer sequence table. Figure S1. CD56 enrichment improves desmin positivity and morphological appearance of tissue engineered constructs. Figure S2. Characterisation of CD56- populations. Table S3. Table of donor sorting efficiencies and yields. Figure S3. Col IV and Laminin localisation in engineered skeletal muscles. Figure S4. Expression of Runx2 and Pparg across recovery. [file 12915_2020_884_MOESM1_ESM.docx]

**Table S1: Individual donor characteristics**

| **Donor** | **Sex** | **Age** | **Muscle identity** |
| --- | --- | --- | --- |
| 1 | Male | 25 | *Vastus lateralis* |
| 2 | Male | 30 | *Vastus lateralis* |
| 3 | Female | 25 | *Vastus lateralis* |
| 4 | Male | 21 | *Vastus lateralis* |
| 5 | Female | 25 | *Vastus lateralis* |

**Table S2: Primer sequence table**

| Gene | NCBI accession no. | Sequence |
| --- | --- | --- |
| POLR2B | NM_000938.3 | Fw: AAGGCTTGGTTAGACAACAG  Rv: TATCGTGGCGGTTCTTCA |
| PAX7 | NM_013945.3 | Fw: ACCCCTGCCTAACCACATC  Rv: GCGGCAAAGAATCTTGGAGAC |
| MYOG | NM_002479.6 | Fw: CAGCTCCCTCAACCAGGAG  Rv: GCTGTGAGAGCTGCATTCG |
| PPARG | NM_015869.5 | Fw: AAAGACAACGGACAAATCAC  Rv: GGGATATTTTTGGCATACTCTG |
| RUNX2 | NM_001024630.4 | Fw: GCAGTATTTACAACAGAGGG  Rv: TCCCAAAAGAAGTTTTGCTG |
|  |  |  |

**Figure S1: CD56 enrichment improves desmin positivity and morphological appearance of tissue engineered constructs. (a)** Phase contrast images of human myogenic precursor cells. Black arrows highlight cells with sort square morphology, red arrows show cells with extended fibroblast like morphology. **(b)** Fluorescent micrographs (10x) of unsorted and CD56 enriched cultures following 6 days in differentiation media. Cultures are stained for desmin (green) and nuclei (blue), scale bar 100µm. Inset graph – desmin positivity (%). **(c)** Fluorescent micrographs (10x) of tissue engineered cross sections. MyHC (green) and nuclei (DAPI) are used to identify myotubes. Scale bar 100µm. Inset shows peripherally nucleated fibre (white arrow), scale bar 25µm.**(d)** Hydrogel deformation expressed as a percentage of size at day 0. **(e)** Average frequency of myotubes of given width within a single cross section of tissue engineered muscle. **(f)** Average myotube cross sectional area (CSA) **(g)** Total myotubes per cross section **(h)** Percentage of total cross sectional area occupied my MytHC structures **(i)** Myotube density expressed as myotubes per mm^2^. All data displayed as mean ± SD with points depicting individual constructs, data collected from n=3 engineered muscles.

**
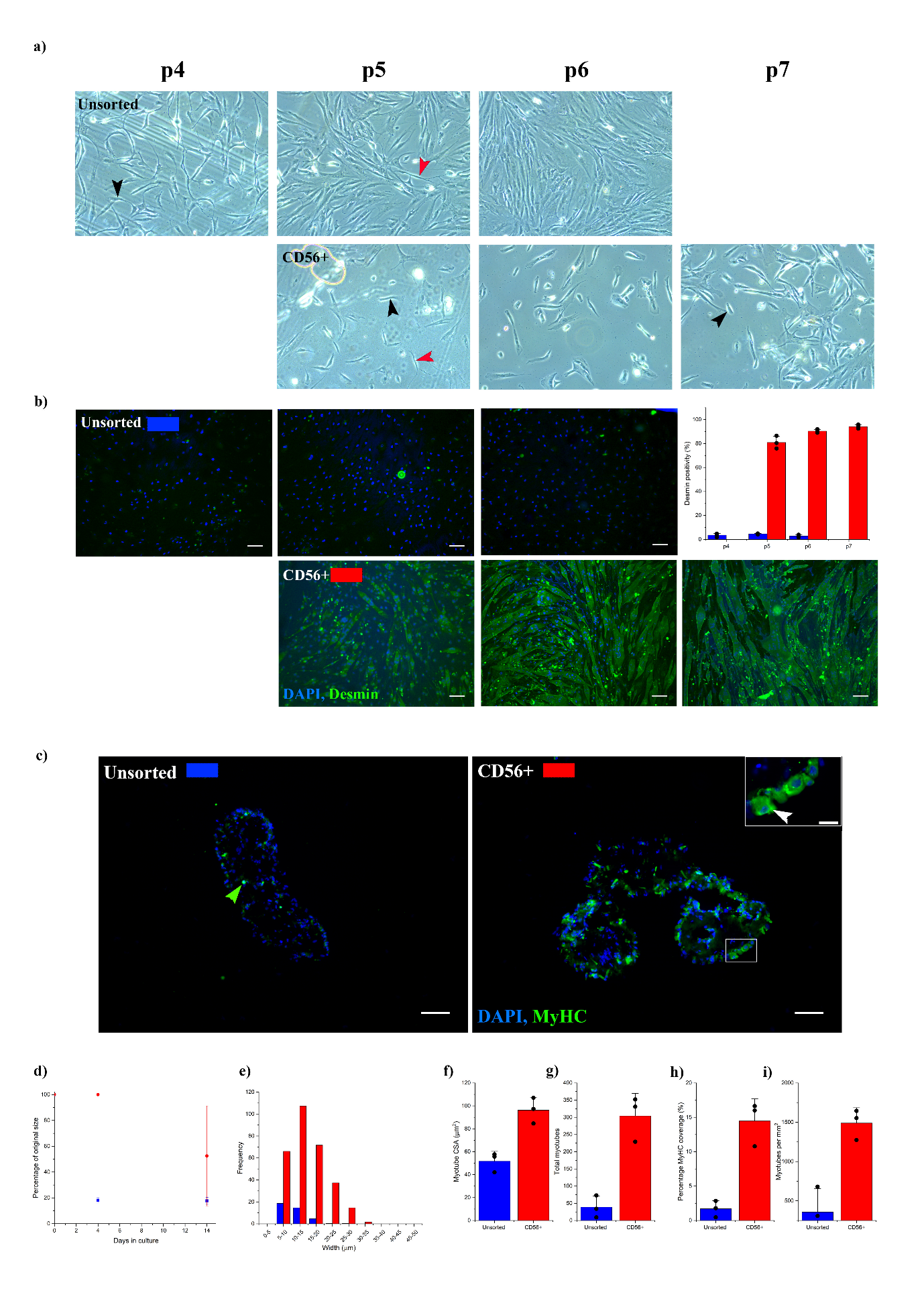
**

**
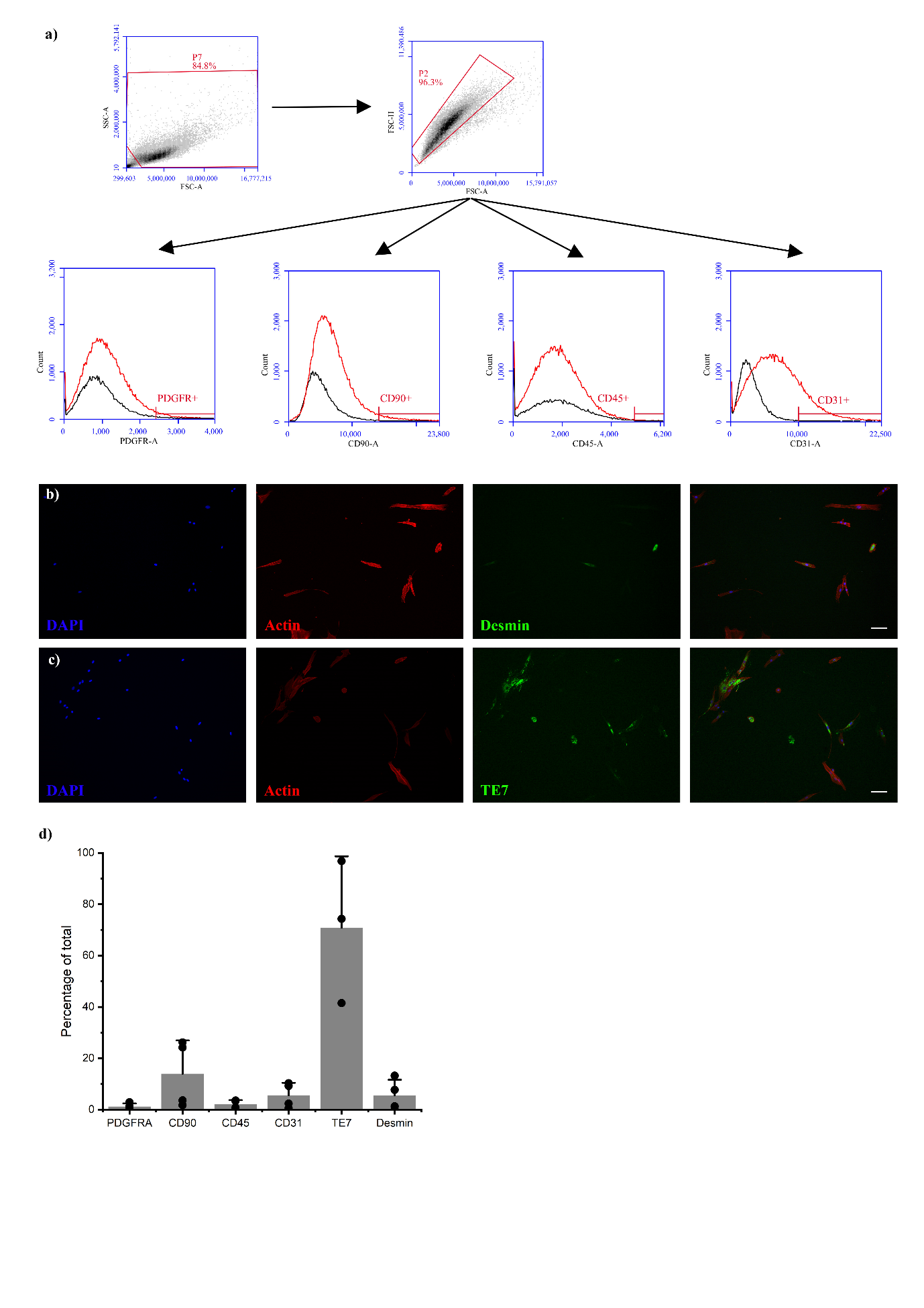
**

**Figure S2: Characterisation of CD56- populations. (a)** Representative flow analysis plots, showing debris removal, doublet discrimination of FMO guided gating. Black lines on fluorescence histograms describe associated FMO while red lines describe stained samples. **(b)** Micrographs showing desmin staining of CD56- cells, scale bar represents 100µm. **(c)** Micrographs showing TE7 staining of CD56- cells, scale bar represents 100µm. **(d)** Quantification of flow and immunohistochemical analysis shown as mean ± SD, individual donors as black points, n=4 from 4 donors.

**Table S3: Table of donor sorting efficiencies and yields**

|  | **Cells sorted (x10^3^)** | **CD56- (x10^3^)** | **CD56+ (x10^3^)** |  | **% yeild CD56-** | **% yeild CD56+** | **Total cell yeild** |
| --- | --- | --- | --- | --- | --- | --- | --- |
| Donor 1 | 600 | 300 | 80 |  | 50 | 13.33333333 | 63.33333333 |
| Donor 2 | 1500 | 500 | 300 |  | 33.33333333 | 20 | 53.33333333 |
| Donor 2 | 800 | 500 | 300 |  | 62.5 | 37.5 | 100 |
| Donor 3 | 900 | 200 | 200 |  | 22.22222222 | 22.22222222 | 44.44444444 |
| Donor 4 | 400 | 100 | 50 |  | 25 | 12.5 | 37.5 |
| Donor 4 | 500 | 50 | 50 |  | 10 | 10 | 20 |
| Donor 5 | 400 | 60 | 30 |  | 15 | 7.5 | 22.5 |
| **Average** | **728.5714286** |  |  |  | **31.15079365** | **17.57936508** | **48.73015873** |
| S.D. | 390.3600292 |  |  |  | 19.01133283 | 10.22347301 | 27.44985006 |

**
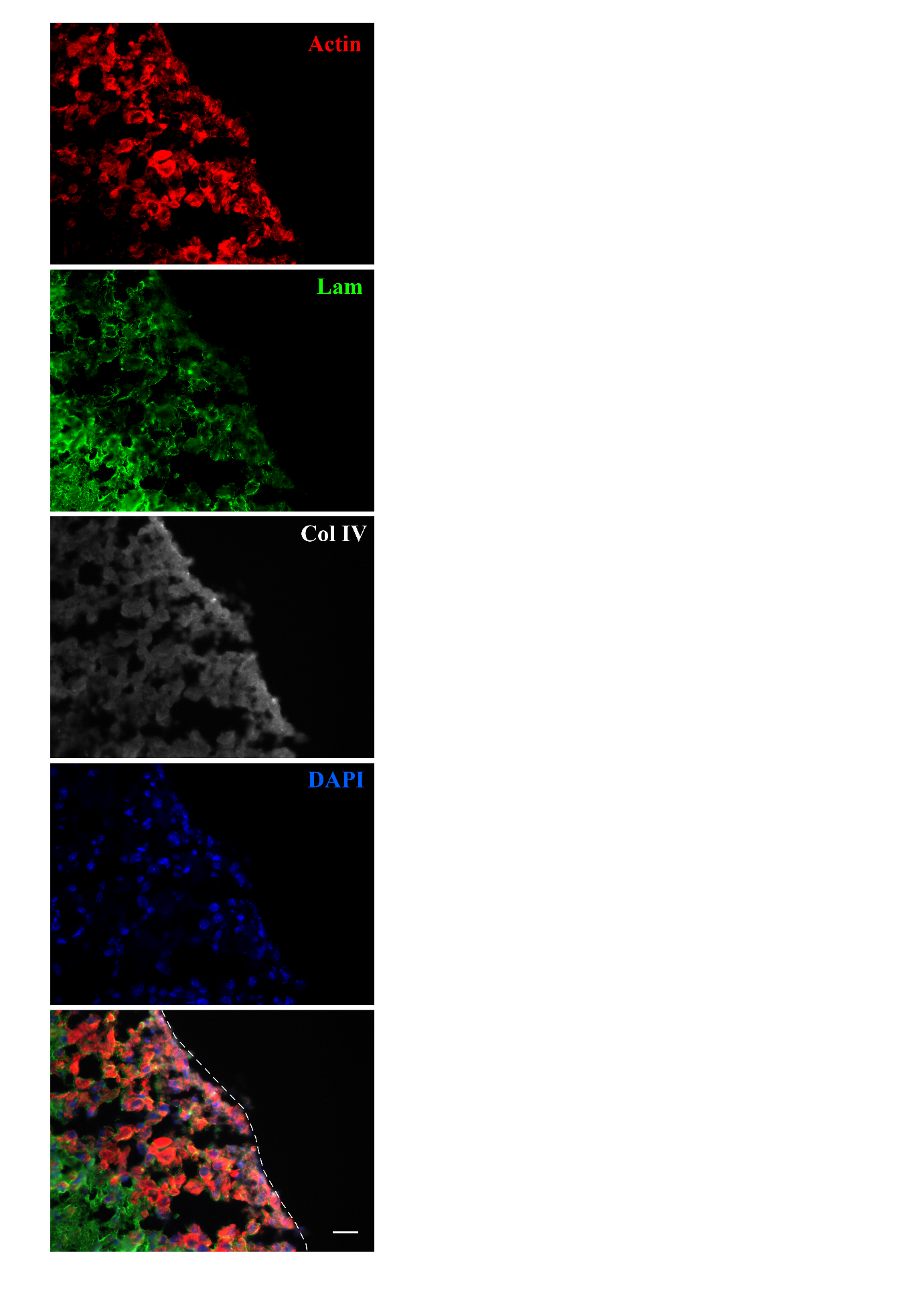
Figure S3: Col IV and Laminin localisation in engineered skeletal muscles.** Micrographs of engineered tissue cross sections stained for actin (red), laminin (green), collagen IV (white) and nuclei (DAPI, blue). Scale bar represents 25µm. White dashed line on overlay image describes the extreme edge of the tissue.

**

**

**Figure S4: Expression of Runx2 and Pparg across recovery.** Graphs show mean ± SD (black bars) and repeat means (grey points). Statistical significance is expressed as ***, p<0.001, n=15 from 5 repeats.
